# Comparison of In-Person and Virtual Implementations of an Obesity Prevention Culinary Nutrition Education Program for Family Care Providers

**DOI:** 10.3390/obesities4030022

**Published:** 2024-08-05

**Authors:** Lenora P. Goodman, Mary M. Schroeder, Kelly Kunkel, Katherine R. Hendel

**Affiliations:** 1 Division of Epidemiology and Community Health, University of Minnesota School of Public Health;; 2 University of Minnesota Extension;

**Keywords:** Children, obesity, nutrition, virtual learning, child care, evaluation, Extension

## Abstract

Start Strong, a 4-week culinary nutrition education, obesity prevention program designed for rural family care providers in low-income areas of Minnesota, was initially an in-person training and recently adapted into a virtual version. Using a quasi-experimental design, this study examined within group and between group (in-person versus virtual) changes in culinary skill confidence and familiarity with food assistance programs after Start Strong participation. Additionally, we examined post-program participant experiences.. The in-person program (n=12, mean age 45 years, September 2019) took place at community locations. The virtual program (n=27, mean age 41 years, Fall 2021–Winter 2022) used online learning and videoconferencing. Following data collection pre- and post-program, we used t-tests to examine within-group changes after Start Strong participation, repeated measures analysis of variance tests to compare outcomes between the in-person and virtual implementations, and Fisher’s exact test to compare post-survey outcomes. The in-person and virtual programs demonstrated similar improvements in cooking skill confidence and familiarity with food assistance programs. Compared to the virtual program, in-person participants reported significantly greater connection with other providers. This evaluation is relevant to addressing disparities in obesity prevention and provides an initial model for public health and community partnerships with ECE providers.

## 1. Introduction

The Institute of Medicine recommends that obesity prevention practices and policies begin in early childhood (before age 5) and include early care and education (ECE) settings, which provide care to nearly 82% of 2–5 year old children in a weekly non-parental care arrangement [1, 2]. Given the role ECE settings play in early childhood health, The Centers for Disease Control and Prevention developed “The Spectrum of Opportunities Framework” to provide states strategies for integrating obesity prevention initiatives into ECE settings [3]. The majority of existing strategies and public health programs aligned with this framework offer professional development and training to ECE child care centers [4]. Child care centers are located at non-residential addresses and care for large numbers of children [5]. However, in the state of Minnesota, 89% of all licensed child care providers are family care providers, which have a maximum capacity of 14 children and are typically located within a residential home [4, 5]. Thus, existing training opportunities overlook the majority of child care providers in the state [4, 5]. University of Minnesota Extension (Extension) developed the *Start Strong: Cooking, Feeding, and More* program to address this gap in education opportunities available for family care providers [4].

Start Strong was a culinary nutrition education program that aimed to prevent obesity by increasing family care providers’ ability to prepare nutritious foods for children and by improving their knowledge of federal food assistance programs [4]. Culinary skills have a role in obesity prevention through their association with increased intake of fruits, vegetables, whole grains, fiber, and vitamins and minerals supportive of a healthy weight [6, 7]. There is some evidence to show that children with caregiver cooking skill and preparation of nutritious food is related to improved child diet quality and lower risk of obesity, and promising evidence that culinary interventions for adults and children can reduce child and adolescent body mass index [7–9]. Generally, community-based culinary obesity prevention programs include cooking skills such as knife skills, meal planning, healthy substitutions, portion size guidance, and techniques for reducing sugar and salt [10–12]. While there is little research examining the role of individual cooking skills in obesity prevention, knife skills tend to be a consistent feature across programs, as they can facilitate saving time, money, and preparation of nutritious foods, potentially reducing reliance on calorie dense convenience foods [6, 13]. To identify additional program components relevant to this population, focus groups with 19 rural family care providers and a statewide survey of ECE providers assessing unmet training needs informed curriculum development [4]. Providers indicated areas they were willing to change and areas in which they needed more education, including learning cooking techniques to use whole grains, adding flavor without salt, and tips to save cost and time [4, 6].

Start Strong curriculum was designed for family care providers in low-income areas that qualified for Tier 1 reimbursement through the Child and Adult Care Food Program (CACFP) [4]. CACFP is a federal program that reimburses the cost of meals to child care centers; centers qualifying for Tier 1 reimbursement provide care to families whose incomes are within 185% of federal poverty guidelines [15]. Obesity disproportionately affects children and families from low-income households, which may have reduced access to nutritious foods [16]. To address this disparity, families within this income guideline qualify for federal food assistance programs, including the Women, Infants and Children (WIC) program, Supplemental Nutrition Assistance Program (SNAP), and free and reduced priced school meals. Child participation in WIC, SNAP, and free and reduced price meals may improve diet quality and reduce obesity prevalence over time [17–19]. Further, the WIC program supports healthy weight in early childhood through additional growth monitoring and nutrition education [20]. Thus, by training providers how to prepare nutritious foods and share information about food assistance programs with families, Start Strong aimed to prevent obesity by improving the early childhood food environment in child care settings and at home [4]. Finally, the Learning Task Model informed Start Strong curriculum design [4]. The Learning Task Model involves learning new information anchored in prior knowledge, practicing new skills, sharing ideas with other providers, and goal setting [9].

Preliminary data evaluating the benefits of participating in the Start Strong program were promising, and family child care providers provided qualitative evidence to support the value of the program [4]. Given that Start Strong was designed for rural family care providers, Extension was interested in developing an online learning format to reduce barriers associated with meeting at a central location. Specifically, compared to urban child care providers, rural family care providers report training location, timing of trainings, and the limited number of available trainings as barriers to receiving additional training [22]. Addressing barriers to training on obesity prevention strategies is pertinent for rural family care providers, as children in rural areas are at higher risk for obesity [23]. Rural communities have played an important role in encouraging the growth of online learning programs to expand regional outreach [24]. Evidence comparing virtual and in-person teaching modalities in the context of higher education suggests there are no differences in knowledge gains and student satisfaction [25]. However, there is a dearth of information available about the implementation of different training modalities for community-based interventions involving rural populations and the type of culinary skills taught in Start Strong.

The onset of the SARS-CoV-2 pandemic provided an impetus to engage family care providers in a virtual format and an opportunity to examine the differences between the in-person and virtual programs through an exploratory quasi-experimental design. Specifically, the aims of this analysis are to (1) examine within group changes after participating in the Start Strong program on key culinary skills and familiarity of food assistance programs; (2) compare the differences in these outcomes between the in-person and virtual iterations of Start Strong over time; and (3) to examine differences in participant experiences from participating in the in-person versus virtual versions of the program. The findings from this analysis will provide initial insight into the performance of the Start Strong intervention using different modalities (in person vs. virtual) and have relevance to the implementation of obesity prevention initiatives in family care centers, particularly among family child care providers in rural regions for whom trainings are often less accessible.

## 2. Materials and Methods

Table 1 provides descriptions of the in-person and virtual iterations of the Start Strong program, including participant recruitment, implementation schemes, and curriculum details. Eligible participants were licensed family care providers from the state of Minnesota who contacted the study team after receiving an email about the study. The in-person Start Strong program took place in September 2019 at a community location determined by Start Strong facilitators. Thirteen participants registered for the course and 12 participants completed all parts of the course (92% participation rate). All 12 participants completed the in-person program, provided complete pre- and post-program data, and were included in the analysis.

The virtual cohort included participants from three separate implementations of the online program: September 2021, October 2021, and February 2022. Virtual participants were required to log into the online Canvas Learning Management System to access course materials and pre- and post-program surveys. Of the 52 participants who initially expressed interest in the online intervention, 12 individuals did not log into the online learning platform to access the intervention materials and 40 completed the training (77% participation rate). However, of these 40 completers, 13 were excluded from analysis for providing either duplicate or incomplete data. The final analytical sample of virtual participants included 27 individuals who completed the training and provided complete pre- and post-program data. A Determination Form was completed for the University of Minnesota Institutional Review Board (IRB, project identification number STUDY00006219). Because this project evaluated a training program (i.e., program evaluation), the IRB determined it was exempt from full IRB review on 16 April 2019.

Survey questions were piloted prior to the 2019 in-person implementation of Start Strong with two child care providers who provided feedback about the length of surveys, sensitivity of survey questions, format of questions, method of delivery, need for clarification, and additional areas for improvement. Extension considered and incorporated this feedback into the pre- and post-program assessments prior to program implementation. Primary outcomes of the in-person and online interventions included confidence about food preparation and familiarity with food support programs, assessed at baseline (pre-program) and immediately after completing the Start Strong program (post-program).

Confidence with food preparation focused on seven key culinary skills addressed throughout the program: using a chef knife, cutting vegetables, cutting fruits, preparing whole grains, using beans and low-cost protein sources, planning menus, and using cooking techniques to reduce salt. Participants rated their confidence with each skill on a 5-point Likert scale (Strongly Disagree to Strongly Agree); “Does not Apply” was also an option.

Participants reported their familiarity with the WIC, SNAP, and free and reduced-price school meals. Response options ranged from 1–4 corresponding to the following possible responses: “Not familiar at all,” “Have heard of the program, but don’t know what it’s about,” “somewhat familiar,” or “very familiar”. Participants were asked how likely they were to refer families to WIC, SNAP, or free and reduced price school meals before and after participating in Start Strong, scored on a 5-point Likert scale (Very Unlikely to Very Likely).

Additionally, after completing the program, participants were asked a series of questions regarding their food purchasing behaviors (i.e., “After attending Start Strong trainings, do you spend more or less money purchasing food for meals and snacks in your child care?” “After attending Start Strong trainings, do you serve more or less healthy foods in your child care?”). Response options included, “Spend more money,” and “More healthy foods,” respectively; and “Spend less money,” and “Less healthy foods,” respectively. “No significant change,” and “Other, please specify” were also options. Participants were asked about their experience with other child care providers during the program (i.e., “After attending Start Strong trainings, are you more connected with other child care providers in your area?” and “Did you learn new ideas from other child care providers as a result of attending the Start Strong trainings?”). Response options to these questions included “Yes,” “No”, “No significant change,” “Not sure,” and, “Other, please specify.” Responses were dichotomized for analysis to “spend less money” or “spend more money,” serve “more healthy foods” or “less healthy foods,” and “yes” or “no,” respectively.

Statistical analyses were performed using STATA 17 Software [27]. Baseline differences between in-person and virtual groups were compared using independent samples t-tests for continuous variables and Fisher’s exact test for categorical variables. After examining baseline characteristics between the in-person and virtual groups, there were no statistically different baseline demographic characteristics between groups. Within group changes after participating in the Start Strong program were assessed using paired samples t-tests. Repeated measures analysis of variance (ANOVA) tests were run to compare outcomes between the in-person and virtual implementations of Start Strong. Responses were analyzed as continuous variables with “Strong Disagree” = 1 and “Strongly Agree” = 5, “Not familiar at all” = 1 and “Very Familiar = 5, and “Very Unlikely” = 1, and “Very Likely” = 5. Post-survey outcome measures were compared using Fisher’s exact test to account for some cells with sample sizes less than five.

## 3. Results

### 3.1. Baseline

Table 2 summarizes participant demographic characteristics. All providers identified as Non-Hispanic White and 95% percent of participants identified as female, which is generally representative of family child care providers throughout the state of Minnesota (nearly all female, average age of 42 years, 94% identify as Non-Hispanic White) [28]. Survey items showed good internal consistency pre-program (Chronbach’s alpha ranging from 0.74–0.92) and post-program (Chronbach’s alpha ranging from 0.87–0.95). Table 3 provides baseline data on in-person and virtual participant confidence with seven key cooking skills, familiarity with food assistance programs, and likelihood of sharing information about food assistance programs with families. The only statistically significant difference at baseline was familiarity with SNAP, which was significantly lower among the in-person group relative to the virtual group (p < .01).

### 3.2. Within Group Changes in Outcomes

Providers in the in-person program significantly improved in confidence with preparing whole grains (p=.02), using low-cost protein sources (p=.02), and using cooking techniques to reduce salt (p<.01, Table 3). The virtual program had significant improvements in all confidence items (p<.05) with the exception of preparing whole grains (p=.12). Providers in the virtual group significantly increased their familiarity with all food assistance programs (p<.05), while providers in the in-person group increased their familiarity with SNAP and free and reduced priced meals (p<.05). Individuals in the virtual program significantly improved their likelihood to share information about all food assistance programs with families (p<.05, Table 3).

### 3.3. Between Group Changes in Outcomes Over Time

Results of the repeated measures ANOVA indicated statistically significant differences between the in-person and virtual groups in familiarity with SNAP (F= 7.29, p = .01). The in-person iteration of Start Strong demonstrated a larger mean increase in familiarity with SNAP (increase of 1.66) than the virtual group (increase 0.78). There were no significant differences between the in-person and virtual iterations of Start Strong over time for any of the remaining outcomes.

### 3.4. Outcomes Assessed at Post-Program

Results of the Fisher’s exact test indicated no significant differences between the in-person and virtual iterations of Start Strong on post-program reports of the amount of money spent on groceries (p=.68), amount of healthy foods served (p=.73), and learning from other childcare providers (p=.47) from participating in Start Strong. The in-person group reported feeling significantly more connected with other childcare providers in their area compared to the virtual group (p<.001) after participating in Start Strong.

## 4. Discussion

The purpose of this study was to determine the preliminary impact of the Start Strong obesity prevention program for rural family care providers, comparing in-person versus virtual implementation modalities, in an exploratory approach using existing data. Specifically, we found that both the in-person and virtual implementations of Start Strong demonstrated significant improvements in using cooking techniques to reduce salt and the virtual group demonstrated significant improvements in familiarity with all food assistance programs. There were only two differences found between the two program modalities. First, there was a greater increase in familiarity with SNAP among the in-person group relative to the virtual group. However, this difference is likely due to significant baseline differences between groups in familiarity with SNAP, as post-program ratings of familiarity were numerically similar (the in-person group increased their familiarity rating from 1.67 to 3.33 and virtual group increased from 2.62 to 3.41). Second, participants in the in-person iteration of Start Strong reported significantly greater connection with other child care providers compared to virtual participants. While identifying revisions that encourage greater connections in the virtual modality requires further exploration, these findings provide initial evidence that both the in-person and virtual implementations of Start Strong can improve cooking skill confidence and increase familiarity with food assistance programs among family care providers.

The similar outcomes between both in-person and virtual modalities found in this study are consistent with previous literature. For example, a randomized control trial using curriculum from the Nutrition and Physical Activity Self-Assessment for Child Care (NAP SACC) found that a web-based, obesity prevention training program was as effective as the in-person program at improving knowledge of child nutrition and physical activity for Child Care Health Consultants, who provide technical assistance to child care centers [29]. Similarly, when comparing the performance of online nutrition courses to in-person courses in higher education settings, online and in-person courses appear equally effective regarding satisfaction with course material and learning outcomes [30]. The online version of Start Strong used a flipped classroom model, in which participants engaged in a learning activity independently (asynchronous) prior to meeting as a group for discussion (synchronous) [31]. Future iterations of the virtual program could benefit from examining how the intervention components perform independently (i.e., synchronous versus asynchronous components) to understand efficacious components of program delivery [32].

Notably, while many of the providers rated their confidence in knife skills and cutting fruits and vegetables relatively high at the start of the program, providers in focus groups reported learning more about these culinary skills as highly desired. Together, these findings may mean that this type of hands-on programming is appealing to providers, but not necessarily an area in need of improvement. However, the inclusion of these skills may facilitate the development of other culinary skills in need of improvement, such as preparing whole grains, using beans and low-cost protein sources, planning menus, and reducing salt when cooking, which can be supportive of a healthy weight [13]. Providers rated these skills lower at baseline and most improved by roughly one full point on a five-point Likert scale. Thus, further research is needed to examine how culinary interventions can support a healthy food environment in child care settings and their effect on children’s diet quality, taste preferences, and health outcomes through exposing care providers to a variety of culinary skills. For example, improving care provider confidence in using cooking techniques to reduce salt could be impactful as intake of sodium-dense foods in childhood is associated with hypertension that can persist into adulthood, and with increased risk of overweight and obesity [33, 34]. While beyond the scope of this study, the improvements in caregivers confidence preparing beans and low cost protein sources could be particularly relevant given current discussions regarding the environmental impact of food choices and plant-based protein options.

Offering in-person and virtual learning options could maximize the accessibility of Start Strong to rural family care providers, and participant preferences for a specific modality is a relevant consideration. For example, some participants may prefer to meet in-person to avoid challenges with internet access and navigating online learning technology, while other participants may find in-person locations inaccessible. Additionally, some rural child care providers may value an opportunity to connect with other child care providers, particularly if they are not aware of other family care providers in their area. A portion of virtual participants interested in participating in Start Strong did not complete the program because they did not log into the online learning system (23%). As use of videoconferencing and online learning platforms becomes increasingly popular, additional research is needed to address technology barriers and literacy, which might prevent some populations from accessing information in online formats. Start Strong videoconferencing sessions employed techniques known to foster community throughout online nutrition interventions by offering weekly group meetings and encouraging group discussions [35]. However, the virtual group did not experience the same connection with other child care providers as the in-person group. The virtual and in-person groups were not significantly different in their ability to learn from other child care providers, suggesting that virtual platforms may better support the exchange of ideas rather than forming connections, particularly for our sample of rural family care providers. It is possible that some providers may benefit from continued connection and education through sharing ideas with each other after the intervention is complete, extending the benefits of participating in the program. Future research is needed to better understand child care provider expectations for making connections with other providers through this type of training, how to improve connections between providers in the virtual format, examine the benefit of connection with other child care providers on long-term outcomes, and investigate how tailored intervention modalities could maximize participant success.

Start Strong curriculum uniquely provides education on food assistance programs. The virtual program, implemented during the SARS-CoV-2 pandemic, resulted in improvements in the likelihood of family care providers recommending WIC, SNAP, and free/reduced-priced school meals to families. This finding could have implications for uptake of food assistance programs by families in need, yet more research is needed to understand how this type of intervention could translate to utilization of food assistance programs. While familiarity with food assistance programs significantly improved, many participants still rated their level of familiarity as “somewhat familiar” instead of “very familiar” soon after being provided substantial information about these programs. Thus, improving education about food assistance programs such that providers feel confident discussing these programs with families after training is an area for future research.

To our knowledge, this is the first study comparing the performance of an in-person versus virtual intervention focused on culinary training among a sample of rural family care providers. The evaluation of Start Strong could have implications for addressing disparities in obesity prevention, as rural children are at greater risk of developing obesity compared to children in urban settings [36]. Further, the findings from this study provide an initial model for public health and community partnerships with ECE providers that expand training opportunities and obesity prevention efforts to reach a larger geographic area. The findings of this study include several limitations. The surveys used to assess outcomes were not designed to make rigorous comparisons, and future programs may benefit from measuring outcomes using standardized measures. The opportunity to compare in-person and virtual implementation of the program arose due changes made following the COVID-19 pandemic. As a result, data for the in-person and virtual implementations of Start Strong were collected at different time points and were taught by different class facilitators. The small sample size further limits our ability to account for potential confounding variables in our analysis. These factors limit the conclusions that can be made regarding differences and similarities between the implementation modalities. Our sample of child care providers includes individuals who identified only as Non-Hispanic White and 95% of providers identified as female. These results are from a convenience sample recruited with the assistance of CACFP sponsors and specifically reached child care providers in rural Minnesota. Further, individuals in our sample chose to participate in this voluntary training opportunity and may have had more interest in nutrition than providers who did not choose to participate. Our sampling method limits the generalizability of our findings to other locations and among family care providers who identify differently, and in combination with our study measures, did not allow for a priori calculation of sample size to detect a desired treatment effect prior to program implementation. Finally, since we do not have data on child growth, we were unable to determine whether Start Strong had an affect on the body mass index of children cared for by the family care providers who participated in the training. Future longitudinal research should examine changes in child growth over time following a culinary obesity prevention intervention, as well as identify specific, essential cooking skills (e.g., knife skills) supportive of a healthy weight.

## 5. Conclusions

Our findings provide promising preliminary evidence that both the in-person and online versions of Start Strong are impactful at improving cooking skill confidence and increasing familiarity in food assistance programs, which are factors related to obesity prevention in the short-term. Notably, by eliminating the need to meet at a central location, the online version is capable of reaching audiences in wider geographic areas, particularly in rural communities, where rates of obesity in children are higher relative to urban areas. However, additional attention to the importance of connection with other child care providers is needed. Further research employing designs that are more rigorous will provide additional insights to beneficial components of the Start Strong program that can be adapted for nutrition education initiatives in other states and child care settings.

## Figures and Tables

**Table 1.** Description of Individual and Shared Features of Start Strong across Program Delivery Modalities.

Program Characteristic	In-Person (Fall 2019)	Virtual (Fall 2021-Winter 2022)
Recruitment	CACFP^1^ sponsors and county child care licensing organizers contacted licensed child care providers via email to share information about Start Strong.. There were an estimated ~5474 licensed family care providers in the state of Minnesota during this study [26]. Although this number of family care providers may have received information about the program at both time points, the number who may have had interest in the in-person implementation of the program was limited by geographic location.	
Incentives for participating	Participants received a $100 gift card to compensate for travel mileage, child care,, and other expenses associated with program attendance.	For completing in the program, participants chose 2 kitchen items worth a combined value of $25 from an online store.
Format of program	Participants met weekly with a facilitator for 2 hours per week at an Extension office and community center, determined by the Start Strong facilitator. Facilitators led group discussions to introduce the curriculum and content of the training. Hands-on experiences, such as food preparation and cooking activities, reinforced concepts and created opportunities for group discussion and reflection. Participants set weekly goals related to curriculum topics. Educational handouts were provided in class.	Participants completed self-paced lessons provided through the learning management software, which included in the curriculum in the form of learning modules and interactive activities. Lessons were open a week prior to being due. To reinforce learning concepts, participants spent additional time independently engaging in the hands-on cooking activities provided to in-person participants. Participants attended 4 scheduled virtual meetings lasting 30 minutes after completing the lesson, which included group discussion led by the facilitator. Educational handouts were mailed to participants.
Facilitator	Two facilitators were assigned to each monthly training. Each month (September 2019, September 2021, October 2021, and February 2022) had a different set of facilitators. At least one facilitator each month had experience teaching Start Strong. All facilitators were Extension educators in Health and Nutrition in the Department of Family, Health, and Wellbeing at the University of Minnesota. All facilitators had experience with adult education and working with child care providers. Their roles included acting as developers of the in-person class and trainers for the online class.	
Curriculum	Week 1: Review of CACFP, Knife skills for cutting vegetables, Equipment to save time and money Week 2: Review of SNAP^2^, Knife skills for cutting root vegetables, Cooking techniques to decease sodium Week 3: Review of WIC^3^, Exploring new choices: whole grains and lower cost protein foods. Week 4 Curriculum: Quick menu ideas, Knife skills for cutting fruit, Menu planning and school meals.	
Benefits for participating	Eight continuing education professional development hours approved by Develop, a Minnesota based organization that accredits trainings for child care providers. Three books about food and nutrition valued at $1 each.	

1 CACFP = Child and Adult Care Food Program;

2 SNAP = Supplemental Nutrition Assistance Program;

3 WIC = Women, Infants, and Children Program

**Table 2.** Demographic characteristics of child care providers; Mean (SD) or count (%).

Characteristic	Full Sample (N=39)	In-Person (N=12)	Virtual (N=27)
Age	42.2 (12.8)	45.2 (10.0)	41.0 (14.0)
Female	37 (95%)	12 (100%)	25 (93%)
Male	2 (5%)	0 (0%)	2 (7%)
Race/ethnicity			
Non-Hispanic/Latino White	39 (100%)	12 (100%)	27 (100%)
Years in child care			
<5	11 (28%)	2 (17%)	9 (33%)
5–15	12 (31%)	2 (17%)	10 (37%)
6–25	7 (18%)	3 (25%)	4 (15%)
26+	9 (23%)	5 (42%)	4 (15%)
Participation in CACFP			
Yes	37 (95%)	12 (100%)	25 (93%)
No	2 (5%)	0 (0%)	2 (7%)
Completion of child nutrition training in the past year that was not food safety or CACFP compliance related			
Yes	14 (36%)	7 (58%)	7 (26%)
No	25 (64%)	5 (42%)	20 (74%)
Number of children cared for annually	9.60 (3.50)	10 (4)	9.4 (3.3)

**Table 3.** Confidence in cooking and familiarity with food assistance programs by program modality; Mean (SD).

	In-Person (N=12)				
Confidence with:^a^	Pre-program	Post-program	Difference	Effect Size	P-value^b^
Using a chef knife	4.08 (.79)	4.25 (1.22)	.17 (1.27)	.13	.66
Cutting vegetables	4.50 (.52)	4.50 (1.17)	0 (1.04)	0	1
Cutting fruit	4.42 (.52)	4.33 (1.23)	.09 (1.08)	.08	.80
Preparing whole grains	3.42 (1.31)	4.50 (1.17)	1.08 (1.31)	.82	**.02**
Using beans and low cost protein sources	3.25 (1.29)	4.33 (1.15)	1.08 (1.38)	.78	**.02**
Planning menus	3.92 (1.00)	4.25 (1.22)	.33 (1.37)	.24	.42
Using cooking techniques to reduce salt	3.00 (.74)	4.33 (1.15)	1.33 (.89)	.01	**<.01**
**Familiarity with food assistance programs:** ^c^					
WIC^1^	3.17 (.83)	3.67 (.49)	.50 (.80)	.63	.05
SNAP^2^	1.67 (.65)	3.33 (.49)	1.66 (.98)	1.69	**<.01**
Free and reduced priced school meals	2.83 (1.19)	3.67 (.49)	.84 (1.03)	.82	**.02**
**Likelihood of sharing information about food assistance programs with families:** ^c^					
WIC	4.42 (.67)	4.83 (.40)	.41 (.79)	.52	.10
SNAP	3.83 (1.27)	4.83 (.40)	1.0 (1.04)	.96	**<.01**
Free and reduced priced school meals	4.33 (.89)	4.75 (.45)	.42 (.79)	.53	.10
	Virtual (N=27)				
Confidence with:^a^	Pre-program	Post-program	Difference	Effect Size	P-value^b^
Using a chef knife	4.19 (1.04)	4.78 (.42)	.59 (1.01)	.58	**.01**
Cutting vegetables	4.41 (.80)	4.78 (.42)	.37 (.88)	.42	**.04**
Cutting fruit	4.52 (.58)	4.81 (.39)	.29 (.67)	.43	**.03**
Preparing whole grains	4.19 (.74)	4.52 (.64)	.33 (1.04)	.32	.11
Using beans and low cost protein sources	3.63 (.97)	4.22 (.70)	.59 (1.01)	.58	**.01**
Planning menus	3.85 (1.06)	4.52 (.70)	.67 (1.33)	.50	**.02**
Using cooking techniques to reduce salt	3.63 (1.00)	4.59 (.50)	.96 (1.16)	.83	**<.01**
**Familiarity with food assistance programs:** ^c^					
WIC^1^	3.11 (.93)	3.63 (.49)	.52 (.80)	.65	**<.01**
SNAP^2^	2.63 (.84)	3.41 (.64)	.78 (.93)	.84	**<.01**
Free and reduced priced school meals	3.07 (1.04)	3.63 (.49)	.56 (.89)	.63	**<.01**
**Likelihood of sharing information about food assistance programs with families:** ^c^					
WIC	4.26 (1.06)	4.74 (.45)	.48 (1.12)	.43	**.03**
SNAP	3.96 (1.22)	4.70 (.54)	.74 (1.35)	.55	**.01**
Free and reduced priced school meals	4.26 (1.06)	4.78 (.42)	.51 (1.09)	.47	**.02**

a Response options ranged from 1–5.

b P-values were determined using paired samples t-tests with significance set at p=0.05. Values in bold indicate statistically significant within group changes.

c Response options ranged from 1–4.

1 WIC = Women, Infants, and Children Program

2 SNAP = Supplemental Nutrition Assistance Program

## Data Availability

The raw data supporting the conclusions of this article will be made available by the authors on request.
